# Feasibility of an Immersive Virtual Reality Intervention for Hospitalized Patients: An Observational Cohort Study

**DOI:** 10.2196/mental.5801

**Published:** 2016-06-27

**Authors:** Sasan Mosadeghi, Mark William Reid, Bibiana Martinez, Bradley Todd Rosen, Brennan Mason Ross Spiegel

**Affiliations:** ^1^ Cedars-Sinai Health System Health Services Research Los Angeles, CA United States; ^2^ Cedars-Sinai Health System Inpatient Specialty Program Los Angeles, CA United States

**Keywords:** virtual reality therapy, hospitalization, feasibility studies

## Abstract

**Background:**

Virtual reality (VR) offers immersive, realistic, three-dimensional experiences that “transport” users to novel environments. Because VR is effective for acute pain and anxiety, it may have benefits for hospitalized patients; however, there are few reports using VR in this setting.

**Objective:**

The aim was to evaluate the acceptability and feasibility of VR in a diverse cohort of hospitalized patients.

**Methods:**

We assessed the acceptability and feasibility of VR in a cohort of patients admitted to an inpatient hospitalist service over a 4-month period. We excluded patients with motion sickness, stroke, seizure, dementia, nausea, and in isolation. Eligible patients viewed VR experiences (eg, ocean exploration; Cirque du Soleil; tour of Iceland) with Samsung Gear VR goggles. We then conducted semistructured patient interview and performed statistical testing to compare patients willing versus unwilling to use VR.

**Results:**

We evaluated 510 patients; 423 were excluded and 57 refused to participate, leaving 30 participants. Patients willing versus unwilling to use VR were younger (mean 49.1, SD 17.4 years vs mean 60.2, SD 17.7 years; P=.01); there were no differences by sex, race, or ethnicity. Among users, most reported a positive experience and indicated that VR could improve pain and anxiety, although many felt the goggles were uncomfortable.

**Conclusions:**

Most inpatient users of VR described the experience as pleasant and capable of reducing pain and anxiety. However, few hospitalized patients in this “real-world” series were both eligible and willing to use VR. Consistent with the “digital divide” for emerging technologies, younger patients were more willing to participate. Future research should evaluate the impact of VR on clinical and resource outcomes.

**ClinicalTrial:**

Clinicaltrials.gov NCT02456987; https://clinicaltrials.gov/ct2/show/NCT02456987 (Archived by WebCite at http://www.webcitation.org/6iFIMRNh3)

## Introduction

For decades, inpatient health care providers have recognized that patient management does not merely entail acute symptom management; hospitalized patients may also experience anxiety, uncertainty, and boredom exacerbated by a radical change in living environment and loss of customary rights and privileges [1]. In order to care for the whole patient, hospitalists must consider not only the biological impact of illness, but also the psychosocial impact. However, the dynamic nature of hospital medicine, coupled with limited time to spend with individual patients, pose challenges to offering holistic inpatient care.

Recent advances in virtual reality (VR) technology offer a compelling opportunity to address inpatient biopsychosocial distress. VR devices provide immersive, realistic, three-dimensional experiences that “transport” users to novel environments. Thus, VR has potential to alleviate negative aspects of hospitalization by providing multisensory information and allowing patients to “escape” to pleasant locations and realities [2].

Previously, VR has been tested in a variety of disease states, including obesity [3-5], anxiety disorders [6-8], pain management [2,9-12], oncology [13], and neurorehabilitation [14,15]. Concurrent improvements in software and hardware design, as well as associated cost reductions, have made VR promising for more widespread use in health care. However, the practicality and qualitative experiences of using VR at scale in the general acute hospital setting has not been formally evaluated in peer-reviewed publications. Because the hospital environment poses unique challenges that outpatient clinics or rehabilitation units do not, it is important to understand the “real-world” practicality of using VR in hospitalized patients—this is a necessary first step before pursuing more extensive evaluation of VR on inpatient outcomes and resource utilization. Thus, we assessed the eligibility, usability, and acceptability of VR equipment and software in a diverse cohort of hospitalized patients in an urban, community-based, academic medical center.

## Methods

### Participants

We screened adults (≥18 years) admitted to the Inpatient Specialty Program at Cedars-Sinai Medical Center in Los Angeles, California, over a 4-month period (August to November 2015). We excluded patients who could not consent, were placed in contact isolation, or had head wounds or bandages that would interfere with the VR goggles. In addition, because VR may cause motion sickness in some users [16], we excluded patients with a history of motion sickness and vertigo, and anyone experiencing active nausea or vomiting. Because there is a theoretical risk of inducing seizures with VR (Samsung Gear user manual cites a 0.025% risk from pediatric data) [17], we also excluded patients with a history of seizures or epilepsy.

### Virtual Reality Hardware and Software

**Figure 1:**
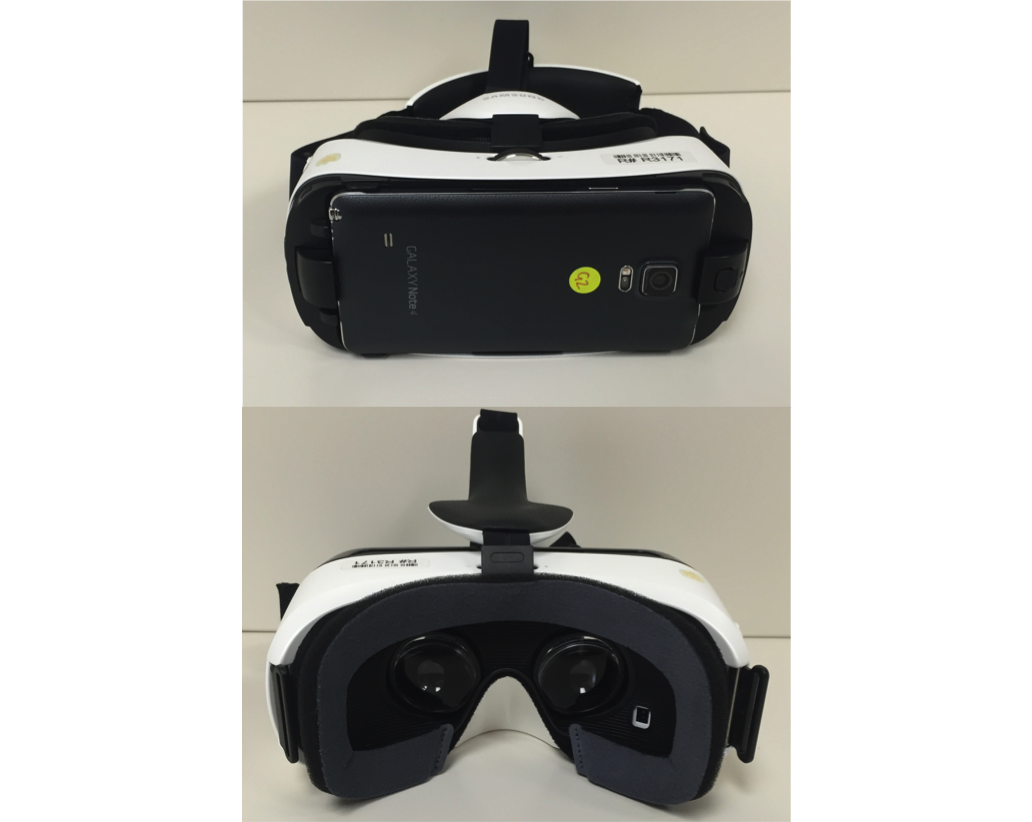
Samsung Gear Goggles.

We used a Samsung Gear VR Innovator edition goggle set, fitted with a Samsung Galaxy Note 4 mobile phone to deliver VR images and sound (Figure 1). We selected this equipment because, at the time of our study, it was a commercially available headset in wide use. In addition, the equipment has minimal visual latency (ie, minimal lag time between head movement and visual tracking) compared to other available form factors, such as Google Cardboard. In consultation with experts in VR health care software (AppliedVR, Los Angeles, CA, USA), we selected four diverse VR software modules: (1) Paint Studio, where users “paint” a picture using head gestures to control the paintbrush; (2) TheBluVR, an underwater ocean exploration; (3) Cirque du Soleil, where users share the stage with performers performing a graceful and harmonious aerial acrobatics while suspended from long, silk bands of fabric; and (4) Tours of Iceland, an aerial tour of rich topographies. These modules were selected because they contain minimal triggers of emotional distress or motion sickness, present a wide range of visual and auditory stimuli, and are considered pleasant experiences by typical users. Each VR experience lasted between 3 and 5 minutes in length.

Prior to patient use, we cleaned fabric surfaces of the Samsung Gear set using Virex, the plastic housing using Sani-Wipes, and the glass lenses using alcohol-based lens cleaner. We placed sanitary disposable fabric covers on the VR goggles for each individual user, and fitted head caps on patients to minimize direct contact with the device—precautions recommended by our infection control department. We briefly instructed patients on the use of the VR goggles, and asked them to watch the four VR experiences in the order preferred by the patient. After each patient completed the study, we discarded the disposable head cap, fabric cover, and foam backing from the device.

### Quantitative Analyses

We collected descriptive data from the health record for each patient, including age, sex, race, ethnicity, and the reason for admission. We recorded VR experience presentation order and any use of corrective lenses. We used chi-square and Fisher exact tests to compare characteristics of patients willing versus unwilling to use VR. All analyses were conducted using Stata version 13.0 (StataCorp LP, College Station, TX, USA)

### Qualitative Analyses

We conducted semistructured cognitive debriefing interviews [18,19] with patients after exposure to VR, asking questions about their overall experience, the VR device, and the software. Before initiating the study, we developed a guide with patient instructions, open-ended think-aloud exercises [18], and scripted probes (Multimedia Appendix 1). For example, the think-aloud exercise instructed patients as follows: “When you think about your experience participating in the VR study, what is the first thing that comes to your mind?” An example scripted probe was: “If a friend or family member asked you about the device, what would you tell them about it?”

A trained moderator led each interview, which lasted approximately 15 minutes. We initially asked patients to describe their experience in their own words and without prompting, followed by scripted probes regarding specific experiences with the VR hardware and software. A social scientist with training in qualitative text analysis (BM) coded responses to the debriefing interview based on themes described by patients (eg, software novelty; device comfort), and presented the results as qualitative findings.

This study was approved by the Cedars-Sinai Institutions Review Board (Protocol #00039751).

## Results

### Patient Selection and Enrollment

**Figure 2:**
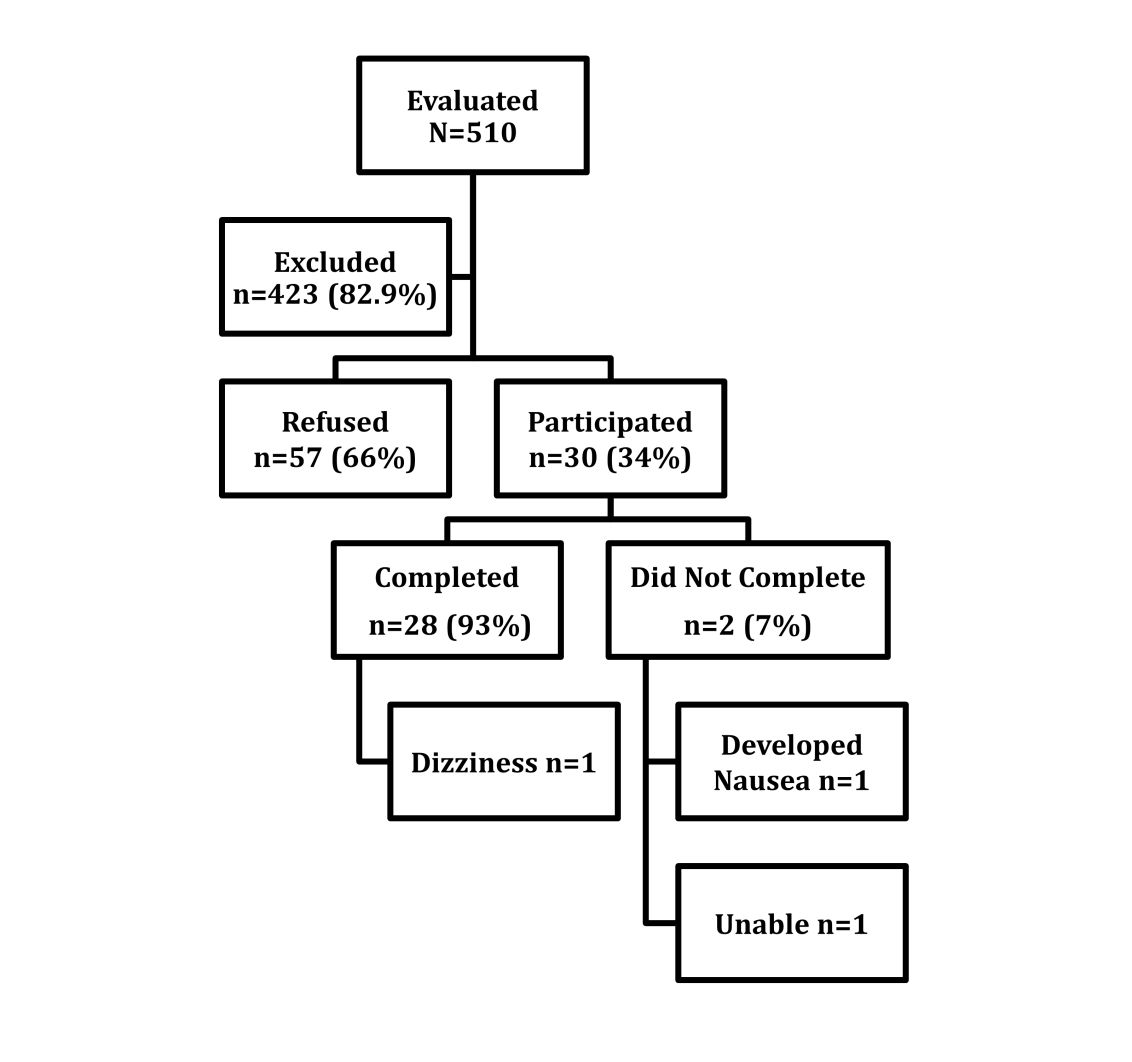
Study flowchart.

Figure 2 presents the flowchart of patient identification and enrollment. We evaluated a total of 510 hospitalized patients for eligibility, of whom 423 (82.9%) failed to meet inclusion criteria. The most common reason for exclusion was presence of one or more neurological diagnoses that either hindered ability to participate or increased risk of a VR adverse event (epilepsy: 6.4%, 27/423; recent stroke: 11.8%, 50/423; dementia: 10.6%, 45/423; other neurological disease: 23.8%, 101/423). Another 26.0% (110/423) of excluded patients were ineligible due to respiratory or contact isolation status. The remainder were excluded because of being too frail/debilitated (4.3%, 18/423), non-English speaking (2.4%, 10/423), unable to consent (0.7%, 3/423), prone to nausea/vomiting/dizziness (5.7%, 24/423), organ transplant (3.3%, 24/423), mechanical ventilation (2.4%, 10/423), and injury to face/neck (2.4%, 10/423).

Of the remaining 87 patients eligible for VR, 57 (66%) refused to participate in the study. Common explanations included not understanding the purpose of VR, feeling anxious about using the goggles, feeling too tired or too ill to participate, concerns about “losing control” of one’s personal environment at a time when control is already limited, and harboring concerns that VR is a “psychological experiment.”

After excluding patients who were ineligible or unwilling to participate, there were 30 remaining participants, of which 28 completed the full VR protocol. Two patients did not complete the study because of VR-related nausea (n=1) or being too frail to support the weight of the goggles (n=1). Of those who completed the study, one patient reported minor and transient dizziness that subsided within minutes of completion. Table 1 provides descriptive statistics of the final 30 patients who used VR.

**Table 1:** Patient characteristics.

Patient characteristic		Patients willing to use VR (n=30)	Patients unwilling to use VR (n=57)	*P*
Age (years), mean (SD)		49.7 (17.4)	60.2 (17.7)	.01
Sex (male), n (%)		19 (63)	28 (49)	.21
**Race/Ethnicity, n (%)^a^**				.47
	Non-Hispanic white	25 (83)	29 (66)	
	Black	3 (10)	9 (20)	
	Asian	1 (3)	1 (2)	
	Hispanic white	1 (3)	4 (9)	
	Other	0	1 (2)	
**Reason for hospitalization, n (%)**				.43
	Gastrointestinal	9 (30)	22 (39)	
	Cardiac	3 (10)	7 (12)	
	Pain control	3 (10)	1 (2)	
	Infectious disease	8 (27)	7 (12)	
	Hematological/Oncological	2 (7)	7 (12)	
	Neurological	1 (3)	1 (2)	
	Pulmonary	1 (3)	2 (4)	
	Rheumatologic	1 (3)	1 (2)	
	Other	2 (7)	9 (16)	

^a^ Race/ethnicity data only available from 44 of the 57 patients in the unwilling group.

### Comparison of Participants and Nonparticipants

Among eligible patients, those who agreed to use VR were significantly younger than patients who refused (mean 49.1, SD 17.4 vs mean 60.2, SD 17.7 years; *P*=.01). There was no statistical difference in sex, race, ethnicity, or reason for admission among patients who were willing versus unwilling to use VR (Table 1).

### Patient Global Experiences With Virtual Reality

Among the 28 patients who completed the full VR experience and responded to the “think-aloud” exercise, 24 responses were coded as positive (86%), two were neutral (7%), and two were negative (7%). Representative examples of positive responses included the following:

Good distraction... welcome distraction...fun detour. Because it’s boring here in the hospital.

It provides a separation from what’s going on. Difficult to verbalize how.

Representative examples of negative responses includes the following:

The headset was uncomfortable and hard to focus with the dial. The nose part was causing me pain, and I could not fully enjoy it.

This was a new experience for me. But I know there are now holograms you can see in front of you, so this technology is already outdated.

When asked: “How did participating in this study make you feel?” 22 responses were positive (79%), three neutral (11%), and three negative (11%). Representative examples of positive responses included the following:

It made me feel good. Really amazing!

Excited to try something new.

Happy. Got away from being here in the hospital. Who wants to be here? It improved my mood.

Representative examples of negative responses includes the following:

It made me feel like I need classes to learn how to operate this thing.

Anxious about getting dizzy during the experience, even though I didn’t feel that way.

I was disappointed.

When asked: “Would you like to participate in a study like this one again?” 22 responses were “yes” (79%). Representative examples of positive responses included the following:

Yes. Definitely. I can see how it would benefit people. I don’t think it will replace drugs, but in mild pain it can work.

Yes, it is an incredible experience. Very alive. Especially for someone like me that can’t walk. The possibilities are endless.

Representative examples of negative responses includes the following:

No, it didn’t impress me. It didn’t change anything. I would like to see it much better, with a better form of focus. It was hard to focus on the images.

No, the goggles were not comfortable. I would consider it again if there were more comfortable goggles.

### Patient Experiences With Virtual Reality Hardware and Software

Regarding the VR hardware, most participants had a positive view about the device (61%, 17/28). However, patients were split on the comfort level; 14 found the device comfortable and 13 found it uncomfortable (one abstained from answering). When asked regarding improvements on the device, most patients requested enhancements in fit, form, and weight, whereas others indicated it was hard to achieve focused images.

Of the four VR modules in the protocol, most patients (57%, 16/28) selected Tours of Iceland as their preferred VR experience; this module was considered by many to be the most “relaxing” and “real life” of the four selections. Conversely, the Paint Studio was selected as the least preferred VR experience; patients noted that this module did not allow adequate control of the paintbrush, leading some to feel more bothered than satisfied with the experience. The majority agreed that all the videos should be longer, but generally less than 10 minutes at a time. Some suggested longer videos, including full-length feature movies.

### Patient Perceptions About Clinical Benefits of Virtual Reality

We asked participants whether they thought VR could affect their level of anxiety or pain. In all, 43% (12/28) of patients believed VR could change anxiety level, although four individuals thought it might worsen anxiety. Most participants (75%, 21/28) believed that the experience could improve pain by means of distraction. Representative quotes included:

It can help with anxiety because it controls the mind.

I’m easily distracted and this helped because I was focusing on the experience and the instructions. My pain medications were due around the time you came in, but the experience improved my pain, and now I don’t feel like I need them right now.

I did have pain, but this was definitely helpful because it made me forget about the pain. It can improve pain by making you forget about it.

It has the ability to take you out of your pain. You still have the pain but you don’t mind it as much.

This was most relaxing to me, especially since I had pain.

## Discussion

Although VR is widely studied in outpatient settings for a variety of pain, affective, neurological, and behavioral conditions [3-13], there is limited experience using VR in the general hospital setting. Given the significant expense of hospital medicine, coupled with the substantial impact of hospitalization on biopsychosocial well-being, it is possible that VR might be effective and cost-effective in managing hospitalized patients. However, before these claims can be made or tested, it is important to first evaluate the pragmatic aspects of using VR in the hospital.

There are two overarching and somewhat contradictory results of this study. First, we found that despite evaluating 510 inpatients for VR, only 30 (5.9%) were both eligible and willing to experience the technology. Strict application of exclusion criteria, including presence of motion sickness, stroke, seizure, dementia, nausea, and isolation status, rendered 82.9% of participants immediately ineligible. Of the remaining eligible patients, 66% refused to participate for a variety of reasons, including anxiety about the technology and high levels of illness severity. In short, despite seeking to apply VR to a cohort, only a small number of patients were ultimately able and willing to participate. Future research should evaluate patient knowledge, attitudes, and beliefs about VR in the hospital, and enumerate specific reasons why some patients are unwilling to use VR.

Second, among participants, most patients found VR to be a positive and pleasant experience. Patients described how VR could ease anxiety, reduce pain through distraction, and provide an “escape” from the confines and boredom of the hospital room. These qualitative results were further supported by endorsement of most participants that they would use VR again if given the opportunity. Common reasons for enjoying the experience were distraction, immersion, being away from the hospital, doing something beyond their means or ability, and the novelty of the experience. In this manner, VR may support the unmet need for patients to virtually “escape” the hospital environment and achieve some degree of normalcy. Of note, our study was not designed to compare VR to other patient engagement technologies, compare it to online forums or other social networks, place it in the larger context of behavior change interventions, or offer a systematic review of digital mental health interventions for inpatients. We solely tested how patients qualitatively experience VR in a hospital. Indeed, a wide variety of new interventions, including augmented reality and mixed reality will soon compete with VR.

Similar to other evolving digital technologies, we found that patients willing to try VR were significantly younger than those who refused. Older individuals can have more difficulty than younger individuals in adopting new technologies—a term described as the “digital divide” that results from variations in self-efficacy and confidence with technology [20-22]. However, although older patients were more hesitant to participate in this VR study, they tended to be less critical of the technology than younger patients were, and most participants enjoyed the experience, independent of age. Based on this observation, encouraging older patients to use VR may offer benefits to some individuals even if there is initial hesitancy to use the technology.

Although most patients described benefits of using VR, there were important limitations identified as well. The goggles were frequently described as too heavy, hard to fit, uncomfortable, and difficult to focus. In addition, because the VR goggles were considered a medical device by our institutional review board, they required meticulous cleaning between patients, application of fresh liners for each use, and provision of a head cap to minimize infection risk. These technical shortcomings may limit the scalability of VR in the hospital and provide opportunities to improve the form factor of current devices. Optimally, a disposable device, such as Google Cardboard goggles or Homido clip-on goggles, could be used to minimize infection risk and logistical concerns, although the current disposable goggles do not yet provide the same immersive experience as higher-end sets.

This study has several important limitations. First, we did not measure the impact of VR on objective outcome measures, including pain level, vital signs, or resource utilization. We believe it is first important to understand and address pragmatic limitations of VR before commissioning large intervention trials. Nonetheless, if the shortcomings identified in this study can be addressed, and if VR can be scaled to a large population, then future research should evaluate the impact of VR on patient outcomes. Second, we excluded a large majority of patients due to preexisting conditions, most commonly neurological disorders that theoretically increase the risk of VR adverse events. Our exclusion criteria were conservative and strict; future research may consider loosening these criteria to allow more patients to participate, particularly given the very low risk of seizures from VR. Third, because members of our research staff were not directly embedded in the immediate hospitalist care team, it is possible that some patients refused to participate merely due to unfamiliarity with the study personnel. If primary caretakers offered VR, then more patients might be willing to try the technology.

The notion of a “Virtualist Consult Service” that offers tailored VR experiences for hospitalized patient is appealing. However, to realize this vision, several intermediate steps will be necessary. Based on this study, we believe that next steps should be to test different goggle sets and form factors, evaluate longer video experiences, offer VR directly through primary providers, and evaluate the impact of VR on both patient reported outcomes (eg, pain, satisfaction scores) and objective outcomes (eg, vital signs). If VR is shown to be pragmatic, scalable, and effective, then we should evaluate its cost-effectiveness and budget impact by monitoring resource utilization (eg, pain medication), length of stay, and readmissions.

## Appendix

Multimedia Appendix 1 Patient debriefing script.

## Abbreviations

VR: virtual reality
