# Supplementary material for: Feasibility of an Immersive Virtual Reality Intervention for Hospitalized Patients: An Observational Cohort Study
Source: JMIR Ment Health. 2016 Jun 27;3(2):e28. doi: 10.2196/mental.5801 (PMC4940605; doi:10.2196/mental.5801)
Supplement: Multimedia Appendix 1 [file mental_v3i2e28_app1.pdf]

Appendix 1. Patient debriefing script.

Patient Interview Script
The Feasibility, Usability and Acceptability of Virtual Reality Use in the Inpatient Setting

We want to thank you for your participation in the study and for agreeing to talk with us today. This interview will take approximately 10-15 minutes, and we will ask you a number of open-ended questions about your thoughts and feelings about wearing the Virtual Reality device and "participating" in the experiences. There are no 'right' or 'wrong' answers to any of these questions: we want to hear about your experience and listen to your opinion, in your own words.

General "Think Aloud" Probe

When you think about your experience participating in the Virtual Reality study, what is the first thing that comes to your mind?

Participating in the Intervention:

Would you like to participate in a study like this one again? Why?

How did participating in this study make you feel?

(***PROBES***: distracted, happy, confused, dizzy, anxious, at ease, etc.)

The VR Device:

If a friend or family member asked you about the device, what would you tell them about it?

How did you feel about the research coordinator watching you while you were using the device?

How comfortable were you wearing the device?

Did you have any concerns about the device while you were using it? If so, what were they?

Did you have any questions about the device while you were using it? If so, what were they?

(Researcher should record if patient asked questions while using it)

Do you have any thoughts on how the device can be improved?

<u>VR Experiences:</u>

Of the ‘experiences’ you watched, which did you like the most? Why?

Of the ‘experiences’ you watched, which did you like the least? Why?

What was your favorite part about watching the ‘experiences’?

What was your least favorite part about watching the ‘experiences’?

What did you think about the duration of the ‘experiences’? Were they too long? Too short?

What would be your ideal ‘experience’ duration? (e.g. how many minutes long would you want them to be)

Did you have any concerns while you were watching the ‘experiences’? If so, what were they?

Did you have any questions while you were watching the ‘experiences’? If so, what were they?

If at all, how do you think the ‘experiences’ can be improved?

What would you like to see in additional ‘experiences’?

<u>Perceived Effects of Participation:</u>

[***IF ANXIETY IS A CONCERN***:]Do you think that wearing the device affected your anxiety level? [***IF YES***:] How so?

[***IF PAIN IS A CONCERN:***] Do you think that wearing the device affected your pain? [***IF YES:]*** How so?

These are all the questions we have for you today. Is there anything we didn’t mention that you would like to discuss?

Thank you again for your participation in this study, and please feel free to contact our research coordinator if you have anything else you would like to discuss with us about this project.
